# Supplementary material for: Sex differences in health care expenditures and mortality after spousal bereavement: A register-based Danish cohort study
Source: PLoS One. 2023 Mar 22;18(3):e0282892. doi: 10.1371/journal.pone.0282892 (PMC10032540; doi:10.1371/journal.pone.0282892)
Supplement: S1 Table — (DOCX) [file pone.0282892.s001.docx]

**S1 Table. Description of the two study designs investigating the association of spousal loss with medical spending and mortality**

|  | **DiD Design** | **Mortality Analysis Design** |
| --- | --- | --- |
| Inclusion Criteria | Persons in the cohort bereaved in 2013-2015 (49% of bereaved population) | All persons entering the cohort, i.e., at 2011-01-01. |
| Sample Size | 38,027 persons | 924,958 persons |
| Matching/Non Bereaved Group | Each bereaved person is matched with a non- bereaved person alive at the index (matched bereavement date) with similar age, sex, affluence index, number of children and comorbidities. | No matching |
| Time Period/Follow-up | Observe bereaved and non-bereaved group two years pre- and one year post-bereavement. | Follow-up of bereaved individuals starts at date of spousal loss, while non-bereaved start at the entry date (2011-01-01). |
| Adjusting/Stratification strategy | Since the two groups are matched on specific covariates mentioned above, analysis is presented as stratified for different age groups and sexes. | Survival analysis is adjusted for affluence index, number of children and comorbidities and presented stratified for different age groups and sexes. The age groups (in 5-year intervals) were constructed based on age at the index date, i.e., a constructed variable being the age at bereavement for the bereaved group and age at the study entry (2011-01-01) for the non-bereaved one. For example, we compare the mortality hazard between bereaved individuals of age 70-74 at bereavement date with that of non-bereaved ones of age 70-74 at 2011-01-01, while also adjusting for affluence index, number of children and comorbidities. |
