# Supplementary material for: Sex differences in health care expenditures and mortality after spousal bereavement: A register-based Danish cohort study
Source: PLoS One. 2023 Mar 22;18(3):e0282892. doi: 10.1371/journal.pone.0282892 (PMC10032540; doi:10.1371/journal.pone.0282892)
Supplement: S2 Table — (DOCX) [file pone.0282892.s002.docx]

**STROBE Statement—Checklist of items that should be included in reports of cohort studies**

|  | Item No | Recommendation |
| --- | --- | --- |
| **Title and abstract** | 1 | (*a*) Indicate the study’s design with a commonly used term in the title or the abstract Page Number : 1,2 |
|  |  | (*b*) Provide in the abstract an informative and balanced summary of what was done and what was found  Page Number : 2 |
| Introduction | | |
| Background/rationale | 2 | Explain the scientific background and rationale for the investigation being reported  Page Number : 3 |
| Objectives | 3 | State specific objectives, including any prespecified hypotheses  Page Number : 3,4 |
| Methods | | |
| Study design | 4 | Present key elements of study design early in the paper  Page Number : 5,6 |
| Setting | 5 | Describe the setting, locations, and relevant dates, including periods of recruitment, exposure, follow-up, and data collection  Page Number : 5,6,7,8 |
| Participants | 6 | (*a*) Give the eligibility criteria, and the sources and methods of selection of participants. Describe methods of follow-up  Page Number : 5,6,7 |
|  |  | (*b*) For matched studies, give matching criteria and number of exposed and unexposed  Page Number : 6 |
| Variables | 7 | Clearly define all outcomes, exposures, predictors, potential confounders, and effect modifiers. Give diagnostic criteria, if applicable  Page Number : 5,6,7 |
| Data sources/ measurement | 8* | For each variable of interest, give sources of data and details of methods of assessment (measurement). Describe comparability of assessment methods if there is more than one group  Page Number : 5 |
| Bias | 9 | Describe any efforts to address potential sources of bias  Page Number : 6,7 |
| Study size | 10 | Explain how the study size was arrived at  Page Number : 5,6,7 |
| Quantitative variables | 11 | Explain how quantitative variables were handled in the analyses. If applicable, describe which groupings were chosen and why  Page Number : 6, 7 |
| Statistical methods | 12 | (*a*) Describe all statistical methods, including those used to control for confounding  Page Number : 6,7 |
|  |  | (*b*) Describe any methods used to examine subgroups and interactions  Page Number : 6,7 |
|  |  | (*c*) Explain how missing data were addressed  Page Number : 5 |
|  |  | (*d*) If applicable, explain how loss to follow-up was addressed  Page Number : 5,7 |
|  |  | (*e*) Describe any sensitivity analyses  Page Number : n/a |
| Results | | |
| Participants | 13* | (a) Report numbers of individuals at each stage of study—eg numbers potentially eligible, examined for eligibility, confirmed eligible, included in the study, completing follow-up, and analysed  Page Number : 9,10 |
|  |  | (b) Give reasons for non-participation at each stage  Page Number : 10 |
|  |  | (c) Consider use of a flow diagram :  Page Number : n/a |
| Descriptive data | 14* | (a) Give characteristics of study participants (eg demographic, clinical, social) and information on exposures and potential confounders  Page Number : 9,10 |
|  |  | (b) Indicate number of participants with missing data for each variable of interest  Page Number : n/a |
|  |  | (c) Summarise follow-up time (eg, average and total amount)  Page Number : 10,13 |
| Outcome data | 15* | Report numbers of outcome events or summary measures over time  Page Number : 13, Figure 3 |
| Main results | 16 | (*a*) Give unadjusted estimates and, if applicable, confounder-adjusted estimates and their precision (eg, 95% confidence interval). Make clear which confounders were adjusted for and why they were included :  Page Number : 13 |
|  |  | (*b*) Report category boundaries when continuous variables were categorized  Page Number : Table 1, Table 2, Table 3 |
|  |  | (*c*) If relevant, consider translating estimates of relative risk into absolute risk for a meaningful time period  Page Number : n/a |
| Other analyses | 17 | Report other analyses done—eg analyses of subgroups and interactions, and sensitivity analyses  Page Number : n/a |
| Discussion | | |
| Key results | 18 | Summarise key results with reference to study objectives  Page Number : 15, 16, 17 |
| Limitations | 19 | Discuss limitations of the study, taking into account sources of potential bias or imprecision. Discuss both direction and magnitude of any potential bias  Page Number : 19 |
| Interpretation | 20 | Give a cautious overall interpretation of results considering objectives, limitations, multiplicity of analyses, results from similar studies, and other relevant evidence  Page Number : 15, 16, 17, 18,19 |
| Generalisability | 21 | Discuss the generalisability (external validity) of the study results  Page Number : 19 |
| Other information | | |
| Funding | 22 | Give the source of funding and the role of the funders for the present study and, if applicable, for the original study on which the present article is based  Page Number : 20 |

*Give information separately for exposed and unexposed groups.

**Note:** An Explanation and Elaboration article discusses each checklist item and gives methodological background and published examples of transparent reporting. The STROBE checklist is best used in conjunction with this article (freely available on the Web sites of PLoS Medicine at http://www.plosmedicine.org/, Annals of Internal Medicine at http://www.annals.org/, and Epidemiology at http://www.epidem.com/). Information on the STROBE Initiative is available at http://www.strobe-statement.org.
