# Supplementary material for: Sex differences in health care expenditures and mortality after spousal bereavement: A register-based Danish cohort study
Source: PLoS One. 2023 Mar 22;18(3):e0282892. doi: 10.1371/journal.pone.0282892 (PMC10032540; doi:10.1371/journal.pone.0282892)
Supplement: Attachment — Submitted filename: Response to Reviewers.docx [file pone.0282892.s003.docx]

**Response to Reviewer**

| No. | **Comments by Editor** | **Response to Editor** | **Change in the revised draft (Tracked Changes Draft)** |
| --- | --- | --- | --- |
| n/a | Thank you for submitting your manuscript to PLOS ONE. After careful consideration, we feel that it has merit but does not fully meet PLOS ONE’s publication criteria as it currently stands. Therefore, we invite you to submit a revised version of the manuscript that addresses the points raised during the review process. Given the extensive comments by the reviewer, I am returning your manuscript for revision.  Once completed the revised manuscript will be sent out to additional reviewers. | We are very pleased to receive comments by the editor and reviewer. We have responded to each of the points raised, as shown below. We believe that the revised draft has been substantially improved. | n/a |
|  | **Comments by Reviewer #1** | **Response to Reviewer #1** | **Change in the revised draft**  **(Tracked Changes Draft)** |
| **1**. | This is an interesting paper on the association between spousal bereavement and Health Expenditures or Mortality and modifications by age and sex with a special focus on sex differences.  Sex differences in mortality in favour of women are well documented in the literature, whereas the finding of increased healthcare expenses is more novel as is also the increased healthcare expenses of pre bereaved women. | We are pleased to see that the reviewer considers findings of this paper as novel. | N/A |
| **2.** | I suggest changing “Sex Dimorphism” to “Sex differences” as “Dimorphism” denotes a trait that occurs in two distinct forms or morphs within a given species and thus leads the thoughts on causal biological effects on the differences which cannot be inferred based on the results (and also change it throughout the text). I also suggest omitting “retrospective” as all cohort studies, in principle, are prospective, and given that it is a Danish registry-based study, the data were collected prospectively. The analysis in cohort studies is always done retrospectively. I guess it is health care Expenditures which is meant as health is no monetary currency. | We fully agree with the reviewer, and have changed the title accordingly by replacing dimorphism in the title and in the text with differences. The wording of calling the study retrospective is also omitted. Health expenditures were changed with health care expenditures. | The corresponding changes are applied throughout the whole manuscript. |
| **3.** | In Oksuzyan et al 2011 no major sex differences in change in health care use (hospitalisations and medicine use (prescriptions) were found, which tends to contradict the findings in the presented study. The study should be mentioned together with the discrepancy, and also acknowledge the findings in the study of an age effect and sex effect (e.g. table 2 in Oksuzyan et al 2011). The discrepancy could e.g., be due to the higher severity of men's diseases being reflected in increased cost (total health care use measured as services codes, i.e., better measure) but not in the number of prescriptions (less accurate measure). | We thank the reviewer for informing us about this specific paper. We consider it highly relevant to our work. We have therefore discussed this paper both in the introduction section, but also discuss the discrepancy with our study, as suggested, in the discussion section. | Introduction section: Line 82-87  Discussion section: Line 376-381 |
| **4.** | To be able to replicate the study, a more precise description of the used registries should be made. I guess that “Sygesikringsregisteret (SSR)” was used for health care expenditures. The Danish Health Data Authority holds the registry, and Statistics Denmark holds a copy. Thus, a reference to a paper describing the registry at The Danish Health Data Authority should be made (e.g. Scandinavian Journal of Public Health, 2011; 39(Suppl 7): 34–37, or similar). Similarly should be done for the rest of the used registries. | We thank the reviewer for this comment. As the methods and nationwide registers used to extract the relevant dataset and expenditures have already been described thoroughly by previous Danish research, we refer the readers the specific scientific articles, which we cite.  Taking into account this comment, we also felt the need to mention in our work, previous statistics describing the completeness of expenditures for the Danish population. | Methods section: Line 146-147  Methods section: Line 150-152 |
| **5.** | How were comorbidities measured? Hospitalisations? medication use? Please give a more precise description. | Similar to the comment above, we cite previous work describing the computations and methods used. | Methods section: Line 146-147 |
| **6.** | The spline-based survival analysis and best-fit model approach make the results for mortality trustworthy. I wonder if it is possible to address how well the difference in differences linear regression model fitted the observed data? | We thank the reviewer for giving us the opportunity to look further into our linear regression models for the difference in differences analysis. We chose to address this by computing the R-squared of our models. We believe the R-squared statistic is widely known and hence useful to a diverse readership. We implemented this into both the Methods (Statistical Analysis) section but also describing the results in the Results section. | Methods section: Line 179-180  Results section: Line 307-310 |
| **7.** | It was not possible for me to see the “S1 Table”? | We appreciate that remark. The S1 Table was and still is placed as item of Supporting Information, with the description being Supporting Table1, and the name: Supporting_Information.docx. Let us know if still the table cannot be found. | N/A |
| **8.** | Results The results are well-presented and easy to follow.  If possible, confidence should be added for each line in figure 1 and figure 2. | We are pleased to see that the reviewer found our results easy to follow. This was a goal of ours.  Confidence bands have now been added for each line in figure 1 and figure 2 to depict the revolving uncertainty. | Fig1, Fig2a, Fig2b |
| **9.** | It is somewhat unclear if sex differences exist between bereaved women and men across ages. | We thank the reviewer for this comment. We had missed making the comparison explicit in our text. Hence, following your remark this has now been added to the manuscript. | Results section: Line 310-316 |
| **10.** | An additional table with differences in differences for post bereavement for the two sexes would make this easier for the reader to understand the results on sex differences (which is in focus given the title). | We assume that the reviewer proposes to show the pooled (both sexes included) difference in differences analysis. We fully agree that this would be informative and useful. Accordingly, Table 2 has now being modified by including the pooled differences. Additionally, we found out that some of the parts in the text describing the expenditures differences across bereavement stages were missing additional statistical testing. To improve our paper and be more robust, we report the differences along with their 95% CIs to support the text. | Table 2  Results section: Line 278, 280,281,285,286 |
| **11.** | Discussion The discussion is well written and gives a comprehensive discussion with suggestions for the observed results.  The hypotheses on reasons for the sex differences and age differences would, however, be more valid given that previous studies that had indicated similar effects were referenced. E.g., when mentioning Vulnerability and Resilience, a paper like Hodes et al. could beneficially be mentioned and referenced to support the hypothesis given (e.g., L384). | We are pleased to see that the reviewer is happy with the way the discussion has been structured.  We thank the reviewer for pointing us to the paper by Hodes et al.The paper has now been mentioned and referenced in the Introduction and Discussion sections. | Introduction section: Line 74  Discussion section: Line 445-447 |
| **12.** | That bereaved women had a lower health care expenditure before bereavement than other women could, as suggested, be attributed to the scenario that older soon-to-be bereaved females tend to ignore their health status to provide caregiving to their spouse. But why is a similar scenario not seen in men? E.g., a hypothesis is that: men do not lose focus on their health when their spouse is sick, while the opposite is true for women. | We indeed observed a different pattern in males when compared to females. While the youngest soon-to-be bereaved males did not have major differences in their expenditures compared with the non-bereaved ones, the oldest ones showed an anticipatory increase in their pre-bereavement spending. We hypothesize that the observed higher pre-bereavement spending in older males could be due to their inability to maintain functional status, provide caregiving (home-care assistance) to their soon-to-be deceased spouse and housekeeping. We note that the lower pre-bereavement spending patterns were mostly observed in females over 75 years of age and not in the youngest. Hence, we decided to form a hypothesis scenario of the contrasting patterns for males of older age for consistency reasons. The implemented sentence is added to the discussion section. | Discussion section: Line 388, 390, 392-394, 411 |
| **13.** | One minor comment:  L322: ” Females but not males had lower healthcare expenditures before experiencing 32bereavement” should be changed to: ”Females but not males had lower healthcare expenditures than not bereaved women before experiencing bereavement” | We thank the reviewer for that remark. It is now changed in the manuscript. | Discussion section: Line 359-360 |
